# Supplementary material for: Causal Relationship between Aging and Anorexia Nervosa: A White-Matter-Microstructure-Mediated Mendelian Randomization Analysis
Source: Biomedicines. 2024 Aug 16;12(8):1874. doi: 10.3390/biomedicines12081874 (PMC11351342; doi:10.3390/biomedicines12081874)
Supplement: Supplementary file 1 [file biomedicines-12-01874-s001.zip › Supplementary Figure/Figure S8/Leaveoneout plot of MR analysis for TL to ubm-b-1645.pdf]

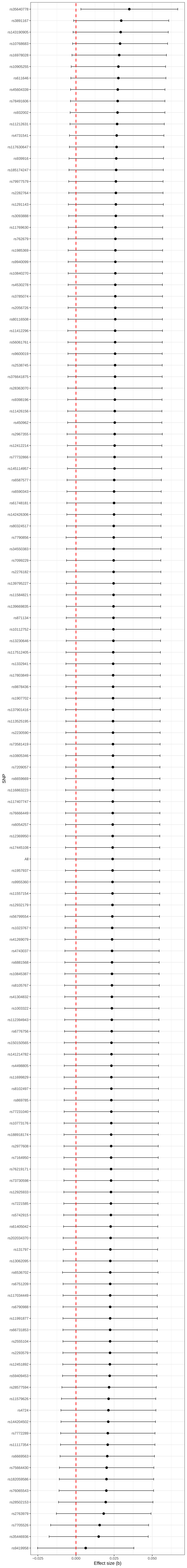
rs35640778
rs3891167
rs143190905
rs10768683
rs16978028
rs10905255
rs611646
rs45604339
rs78491606
rs932002
rs11212631
rs4731541
rs117630647
rs939916
rs185174247
rs79977579
rs2282764
rs1291143
rs3093888
rs11769630
rs762679
rs1985369
rs9940099
rs10840270
rs4530278
rs3785074
rs2056726
rs80116508
rs11412296
rs56061761
rs9600019
rs2538745
rs376641875
rs28363070
rs9398196
rs11426156
rs450962
rs2967355
rs12412214
rs77732866
rs145114957
rs6587577
rs6590343
rs61748181
rs142426306
rs80324517
rs7790856
rs34550383
rs7099229
rs2276182
rs139795227
rs11584821
rs139669835
rs871134
rs10112752
rs13230646
rs117512405
rs1332941
rs17803849
rs9878436
rs1907702
rs137901416
rs113525195
rs2230590
rs73581419
rs10805346
rs7209057
rs6659669
rs116863223
rs117407747
rs76666449
rs6054257
rs12369950
rs17445108
All
rs1957937
rs9955360
rs11557154
rs12932179
rs56799554
rs1023767
rs41269079
rs4743037
rs6881568
rs10845387
rs8105767
rs41304832
rs1003322
rs112394943
rs6776756
rs150150565
rs141214782
rs4498805
rs11699829
rs8102497
rs869785
rs77231040
rs10773176
rs188918174
rs2977608
rs7164950
rs76219171
rs73730598
rs12925933
rs7221585
rs5742915
rs61405042
rs202034370
rs131797
rs13062095
rs6536702
rs6751209
rs117034449
rs6790988
rs11991877
rs66731853
rs2555104
rs2293579
rs12451892
rs59409453
rs28577594
rs11579626
rs4724
rs144204502
rs7772289
rs11117354
rs6669563
rs75664430
rs182059586
rs76065543
rs28502153
rs2763979
rs7705526
rs35446936
rs9419958
SNP
−0.025
0.000
0.025
0.050
Effect size (b)
